# Bacterial community structure of the sunflower (*Helianthus annuus*) endosphere

**DOI:** 10.1080/15592324.2021.1974217

**Published:** 2021-09-30

**Authors:** Bartholomew Saanu Adeleke, Ayansina Segun Ayangbenro, Olubukola Oluranti Babalola

**Affiliations:** Food Security and Safety Niche Area, Faculty of Natural and Agricultural Sciences, North-West University, Mmabatho, South Africa

**Keywords:** Agricultural sustainability, bacterial diversity, culture-independent techniques, amplicon sequencing

## Abstract

Agrochemical applications on farmland aim to enhance crop yield; however, the consequence of biodiversity loss has caused a reduction in ecological functions. The positive endosphere interactions and crop rotation systems may function in restoring a stable ecosystem. Employing culture-independent techniques will help access the total bacteria community in the sunflower endosphere. Limited information is available on the bacteria diversity in sunflower plants cultivated under different agricultural practices. Hence, this study was designed to investigate the endophytic bacterial community structure of sunflower at the growing stage. Plant root and stem samples were sourced from two locations (Itsoseng and Lichtenburg), for DNA extraction and sequenced on the Illumina Miseq platform. The sequence dataset was analyzed using online bioinformatics tools. Saccharibacteria and Acidobacteria were dominant in plant roots, while the stem is dominated by Proteobacteria, Bacteriodetes, and Gemmatimonadetes across the sites. Bacterial genera, *Acidovorax, Flavobacterium, Hydrogenophaga*, and *Burkholderia-Paraburkhoderia* were found dominant in the root, while the stem is dominated by *Streptomyces*. The diverse bacterial community structure at phyla and class levels were significantly different in plant organs across the sites. The influence of soil physical and chemical parameters analyzed was observed to induce bacterial distribution across the sites. This study provides information on the dominant bacteria community structure in sunflowers at the growing stage and their predictive functions, which suggest their future exploration as bioinoculants for improved agricultural yields.

## 1. Introduction

The understanding of the spatial distribution of bacteria communities in plant organs has provided various opportunities in harnessing their potential in agricultural systems.^1^^,2^ Diverse microbial communities colonize plant organs to maximally induce adaptive responses in the plant through various metabolic pathways.^3,4^ Characterization of bacterial endophytes from various environmental samples is known and widely adopted in environmental studies,^5^ although most microbes have not been cultured and isolated. More significantly, insights into microbial biotechnology have unveiled the use of amplicon metagenome sequencing in microbiological studies.^6^ Furthermore, the use of 16S rRNA gene sequencing in evaluating bacterial community structure in the sunflower endosphere is advancing compared to the culture-dependent techniques.^7^

Bacteria that colonize the endosphere could be beneficial, but most do not affect plant fitness.^8^ However, limited information is available on the diversity of endophytic bacterial communities in oil food crops, such as sunflower; thus, necessitating this study. Sunflower is an oilseed crop cultivated in most countries of the world.^9^ Many countries, such as Turkey, Russia, Argentina, Ukraine, and South Africa have tapped into maximizing its potential usage for both industrial and domestic use.^10^

On a large-scale production, reports on the plant growth-promoting microbes associated with sunflower in Southern Africa for improved yield are understudied, possibly due to limited study on sunflower using the next-generation sequencing approach.^11^ Promisingly, the researcher-farmer synergism in sunflower production can provide a lasting solution to food insecurity and shortfall in the global cooking oil supply. More importantly, the use of bioinoculants from bacterial sources has been employed as a potential tool for sustainability of agricultural production systems to meet the world food supply.^12^ As such, working toward devising agriculturally friendly approaches to explore below-and-above ground endophytes is promising in improving crop productivity.^13^ The plant genotype, age, soil type, locations, agricultural practices, and limiting soil nutrients can influence plants’ physiological patterns and their associated bacteria community structure.^14^ Recent findings have revealed the use of culture and non-culture approaches in assessing bacteria community structure in the stem, root, leaves, and seed endosphere of most oilseed crops and medicinal plants.^15–18^ A study by^19^, on the metagenomics profiling of endophytic bacteria from maize has recommended further exploration of endophytic microbes in agriculture, based on their promising effects in shaping plant community, modulating plant ecology, fitness and evolution, and exhibiting strong effects on the biological activities of the plants against pathogens. Interestingly, endophytic microbes have been studied to enable a complete understanding of the mechanism of applications and ensure a thorough assessment of the current state in the knowledge of the microbes in agricultural biotechnology.^20^

In this study, we hypothesized that environmental variables analyzed may induce varied influence on the root endosphere bacterial structure compared to stem endosphere across different locations. Presumptively, these hypotheses can be determined considering the soil physical and chemical parameters in shaping the bacterial community structure in the plant root rhizosphere.^21^ Most bacteria thrive well close to neutral or slightly acidic environments, as low pH (below 4) impedes their growth.^22^

The culture-based techniques in the study of bacteria associated with sunflower have been employed in identifying notable plant growth-promoting bacteria.^23^ Additionally, the use of culture-dependent techniques for detecting identifiable sunflower root-associated endophytic bacteria has been reported with great promise in agriculture.^24,25^ A study by^26^, reported the use of 16S rRNA gene sequencing in the identification of phosphate-solubilizing bacteria for enhanced sunflower growth and improved yields. Evaluation of structural diversity of sunflower endophytic bacteria and comparative analysis of root and stem endosphere bacterial communities across different sites under different agricultural practices have not been documented. Therefore, this study was designed to present the sunflower root and stem-associated bacteria and their predicted functions under different agricultural practices using 16S rRNA gene sequencing. Hence, the comparison of sunflower bacterial endophytes in plant organs will significantly reveal their potential traits and possible applications for improved agricultural production.

## 2. Materials and methods

### 2.1 Site location, sampling, and climatic conditions

In February 2020, samples of sunflower plants (roots and stem) of the same cultivar PAN 7160 CLP were sourced from different sites in Itsoseng (IT) (S26°3′20.106′′E25°56′24.234), Thusong District, and Lichtenburg (LT) (S26°4′31.266′′ E25°58′44.442), Ditsobotla District, North West Province (NWP) of South Africa. A total of 12 samples each for stem and root were collected in triplicate from 4 points from each site. Samples were obtained, aseptically excised, and placed in sterile zip lock bags, kept on ice, and immediately transported to the Microbial Biotechnology Research Laboratory (North-West University, South Africa), and stored at −20°C before use. A 50 g sunflower rhizosphere soil in triplicate from each sunflower across the sites was collected aseptically at the depth of 15–20 cm. Generally, the geographical information in NWP has a record of the annual rainfall of 600 mm, low-level area, trees, grassland, and a temperature range from 17°C to 31°C during summer and from 3°C to 21°C during winter. The historical background of the farmland in Lichtenburg revealed that, for more than 4 decades, sunflower had been cultivated with other crops, such as maize and soybean, on a rotational basis. Water supply is by rainfall and irrigation. The major farm activities include clearing, tilling, ridging, minimal chemical fertilizers (NPK 15:8:4) application, and herbicides (Judo 50EC, Metagon Gold) application to the soil before and after planting. Foliar insecticide spray was applied after plant germination. The farm size is 234 hectares with 6 hectares of sunflower plantation coverage. In Itsoseng, the farm size is 3,500 hectares with 100 hectares of sunflower plantation coverage. Only sunflower is cultivated on the farmland. Urea and organic manure are employed as soil amendments.

### 2.2 Root cleaning, and surface sterilization

Samples were detached with a sterile scalpel, and washed 3 times with sterile distilled water, and allowed to drain for 10–20 minutes. The samples were cut into miniature sizes (1 cm). The surface sterilization was performed following the modified method of ^27^. Briefly, the washed roots and stem were immersed in 70% ethanol for 3 minutes, then in 3% hypochlorite solution for 3 minutes, and finally immersed in 70% ethanol for 30 seconds. The sterilized samples were rinsed 5 times with sterile distilled water and allowed to dry under controlled conditions in a laminar flow cabinet [FILTA-MATIX]. The level of sample sterility was validated according to the method described by ^28^. A few surface-sterilized samples were gently placed in 10 ml sterile distilled water and gently stirred for 1 minute. An aliquot of 0.1 ml suspension was inoculated on Luria Bertani [LB] agar plates, incubated at 28°C for 8–10 days, then assessed for colony growth daily. No bacterial growth formation on the plates validates the removal of root surface bacterial colonizers, and sunflower roots and stem were used for DNA extraction.

### 2.3 DNA extraction process and 16S rRNA gene amplicon sequencing

The extraction of genomic DNA from the surface-sterilized samples was conducted according to the method described by^27^. The surface-sterilized samples were immediately macerated manually in a sterile mortar and pestle. DNA was obtained from 1 g of the macerated sample using commercial DNA kits [DNeasy® Plant Mini kit, Qiagen, USA] following the manufacturer’s protocol and then stored at −20°C before sequencing. The 16S rRNA gene variable V4 region of the amplicon sequence was sequenced on the Illumina Miseq platform. The PCR primers; 515 f/806 r with a definite barcode on the forward primer were used as described by^29^. The PCR was performed in a 30 cycle (5 cycles used on PCR products] using HotStar Taq Plus Master Mix Kit (Qiagen, USA) under the following conditions: 94°C for 3 minutes, followed by 30 cycles of 94°C for 30 seconds, 53°C for 40 seconds and 72°C for 1 minute, and final elongation step at 72°C for 5 minutes. The success of the PCR amplification process and band size were checked in 2% agarose gel. PCR products were purified using calibrated Ampure XP beads following the instructions of the manufacturer. The purified PCR product was used to prepare the DNA library, following the DNA library preparation kit protocol. Sequencing of the extracted DNA was done at MR DNA LP Research Laboratory (Shallowater, TX, USA). For accuracy and sensitivity, a fluorescence Quant-iT PicoGreen dsDNA kit (Invitrogen, Carlsbad, CA, USA) was used to quantify the concentrated dsDNA. The accurate, easy, and quick fluorescence measurement was determined using a dual-channel and compact DQ 300 Fluorometer (Hoefer Scientific Instruments, San Francisco, CA, USA).

Sequence read processing was done using Quantitative Insights Into Microbial Ecology (QIIME 2) 16S pipeline (version 2020.11)^30^ implemented on Nephele microbial bioinformatics platform (version 1.8) (https://nephele.niaid.nih.gov/).^31^ The preprocessing steps included read pair joining using default parameters (% maximum difference of 25, and a minimum overlap of 10), removal of reads with an average Phred score of ≤ 20, removal of chimeras using VSEARCH,^32^ and clustering, using Open Reference Method and SILVA 99 version 132.^33^ Taxonomy assignments were done against SILVA version 132, with a default sequence similarity threshold of 0.99. The chimeric sequences, such as mitochondria, chloroplast, and singleton reads were removed.

### 2.4 Determination of physical and chemical parameters of the sunflower rhizosphere soil

To determine soil physical and chemical parameters, soil tightly associated with the root of sunflower was collected into sterile plastic bags. Triplicates of four sunflower rhizosphere soils were collected from each site. Soil samples from each site were analyzed separately. The soil was air-dried into a fine powder and sieved through a mesh size 2 mm after removing rubble, specks of dirt, stones, and other organic debris. The soil parameters considered include soil pH, nitrate, ammonium, total soil mineral elements (carbon, calcium, phosphorus, potassium, nitrogen, sodium, magnesium), and organic matter. The soil particle size was classified following the methods described by^34^. The measurement of soil pH was performed by dipping Jenway 3520 pH-meter (Cole-Parmer Instruments, Staffordshire, United Kingdom) into a soil suspension containing mixed sieved soil sample and sterile distilled water (1:2) ratio.^35^ The moisture content (MC) was determined based on the dry mass of the soils after oven-dry at 105°C overnight using an oven (Anatech Instruments, Thermo Scientific Inc., SA). The soil total nitrogen and carbon were determined appropriately using the dry combusting technique as described by.^36^ Similarly, the potassium chloride (KCl) extraction method was used to determine the nitrate content.^37^ The organic carbon content was determined according to the methods described by^38,39^. The soil mineral elements, such as calcium, potassium, sodium, calcium, and magnesium contents were determined after the extraction was performed using 1 M ammonium acetate adjusted at pH 7. Subsequently, sodium, calcium, and magnesium present in the soil were determined using an atomic absorption spectrophotometer (Anatech Instruments, Thermo Scientific Inc., SA).^40^ The phosphorus and potassium contents were measured using a flame photometer spectrophotometer (Anatech Instruments, Thermo Scientific Inc., SA), respectively.^40^

### 2.5 Data analysis

The graphical representation of bacteria relative abundance was determined using Shinyheatmap (version 0.12.2).^41^ The differences in the bacterial community structure across the sampling sites were assessed using a Kruskal–Wallis test in the paleontological statistics software package (PAST version 3.20).^42^ The alpha diversity indices, Evenness, Simpson, and Shannon derived from PAST software were used to estimate the diversity and richness of the endophytic bacteria in the samples across sites. Principal coordinate analysis (PCoA) based on a Bray–Curtis dissimilarity matrix was used to determine beta diversity. The distribution of bacteria in the samples across sites for principal component analysis (PCA) plot was performed using the Bray–Curtis distance matrix. PCoA and PCA plots were designed using CANOCO version 5 (Microcomputer Power, Ithaca, NY, USA) software. The canonical correspondence analysis (CCA) was determined on CANOCO software. CCA was employed to assess possible correlations between bacterial communities and measured environmental variables. The predictive functional annotation of the bacterial endophytes in each site was performed on Phylogenetic Investigation of Communities by Reconstruction of Unobserved States (PICRUSt), and the resulting output from different levels, i.e. first, second, and third regarding their predicted functions were obtained.

## 3. Results

### 3.1 Sunflower soil analysis

The physical and chemical analysis of sunflower rhizosphere soil is shown (Table 1). Soil analysis showed higher nitrate values in L (sunflower rhizosphere soil samples from Lichtenburg) (14.15 mg/kg) than I (sunflower rhizosphere soil samples from Itsoseng) (6.44 mg/kg). The soil pH value was neutral at site L and had low acidity at the site I. It was observed that ammonia, phosphorus, magnesium, total carbon, total nitrogen, and clay were higher in site L than in site I.

**Table 1.** Physical and chemical soil analysis

Site	L	I
Organic matter (%)	2.02 ± 0.03 ^c^	1.36 ± 0.05 ^c^
Nitrate (N-NO_3_) (mg/kg)	14.15 ± 0.06 ^h^	6.44 ± 0.04^e^
Ammonium (NH_4_) (mg/kg)	6.04 ± 0.05^e^	5.08 ± 0.08^d^
pH	7.05 ± 0.05 ^g^	6.36 ± 0.05^e^
Resistivity conductivity (ohm)	1045.03 ± 0.02°	2170.03 ± 0.03 ^m^
Phosphorus (P) (mg/kg)	74.93 ± 0.05 ^j^	26.40 ± 0.02 ^h^
Calcium (Ca) (mg/kg)	973.02 ± 0.02 ^n^	424.02 ± 0.17 ^l^
Magnesium (Mg) (mg/kg)	187.02 ± 0.02 ^m^	122.06 ± 0.28^k^
Potassium (K) (mg/kg)	181.03 ± 0.03 ^l^	113.01 ± 0.02 ^j^
Sodium (Na) (mg/kg)	6.51 ± 0.01 ^f^	7.17 ± 0.03 ^f^
Total carbon (%)	0.63 ± 0.04^b^	0.42 ± 0.03^b^
Total nitrogen (%)	0.06 ± 0.01^a^	0.04 ± 0.01^a^
Sand (%)	77.03 ± 0.03^k^	77.02 ± 0.02^i^
Silt (%)	3.01 ± 0.02^d^	5.01 ± 0.01^d^
Clay (%)	20.01 ± 0.02^i^	18.02 ± 0.02 ^g^

**Key**: L = sunflower soil samples from Lichtenburg, I = sunflower soil samples from Itsoseng. Mean±standard deviation having different letters of triplicate determinations are significantly different at P ≤ 0.05 according to DMRT.

### 3.2 Root surface sterilization and sequence data analysis

**Figure 1.**
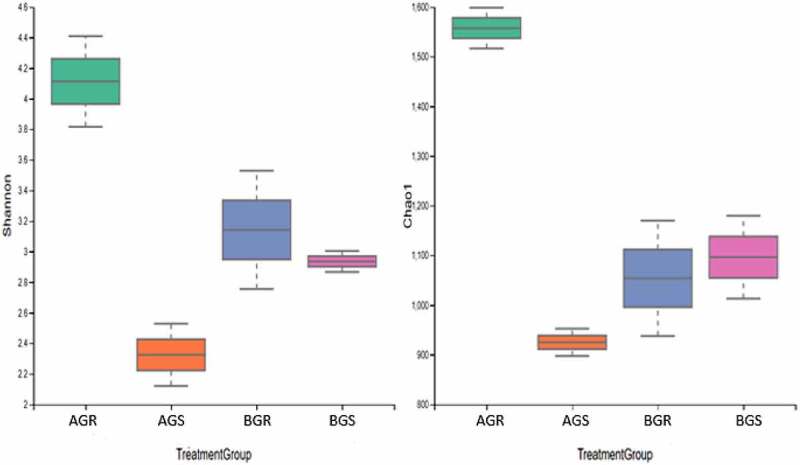
Species diversity. Keyword: AGR – root samples from Itsoseng, AGS – stem samples from Itsoseng, BGR = root samples from Lichtenburg, BGS – stem samples from Lichtenburg.

Taxonomy assignments were done against the SILVA database. The differences in alpha diversity indices in the samples across sites were calculated as Shannon, Simpson, and Evenness (Table 2). At phylum and class levels, a significant difference (*p* < .05) was observed, while at the genus level, no significant difference (*p* > .05) was observed in the bacterial diversity. The highest alpha diversity of endophytic bacteria in the root and stem of growing sunflower based on Shannon indices were attained in BGR (sunflower root from Lichtenburg), followed by AGR (sunflower root from Itsoseng) at class, and the least Shannon index from BGS (sunflower stem from Lichtenburg) and AGS (sunflower stem from Itsoseng) were obtained at the phylum level (Table 2). At the phylum, class, and genus levels, high Shannon (H) index values were obtained in the samples compared to other indices measured across the sites (Table 2). The Chao 1 and Shannon species diversity indices at taxonomic level presented in (Figure 1) showed the differences in the bacteria diversity in the samples across the sites (Itsoseng and Lichtenburg).

**Table 2.** Alpha diversity of endophytic bacteria in the root and stem of growing sunflower

Bacterial level	Indices	AGR	AGS	BGR	BGS	p-value
**Phylum**	Evenness	0.1986	0.1891	0.223	0.2089	0.000006
	Simpson	0.3517	0.1613	0.3927	0.2721	
	Shannon	0.6859	0.4142	0.8021	0.6311	
**Class**	Evenness	0.4231	0.3015	0.452	0.3518	0.0001
	Simpson	0.7184	0.4398	0.7185	0.5611	
	Shannon	1.442	0.9983	1.508	1.258	
**Genus**	Evenness	0.4069	0.2443	0.4423	0.2481	0.5435
	Simpson	0.6797	0.382	0.6787	0.3978	
	Shannon	1.404	0.8931	1.487	0.9088	

*P* values were obtained through the Kruskal-Wallis test. AGR – root samples from Itsoseng, AGS – stem samples from Itsoseng, BGR = root samples from Lichtenburg, BGS – stem samples from Lichtenburg.

**Figure 2.**
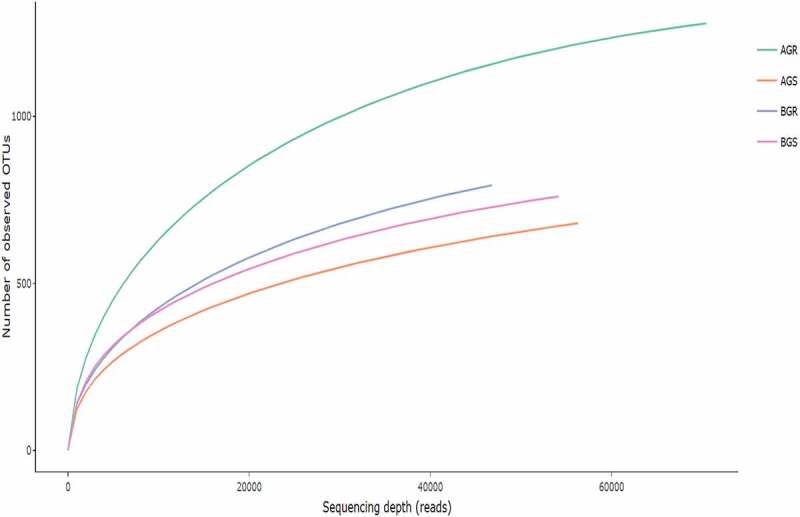
Rarefaction curves of evaluated richness in the root and stem of a sunflower. The vertical axis represents the number of OTUs estimated after sampling, while the number of sequence reads is represented on the horizontal axis.

The total number of input sequences (TNIS) varied across the sites (Table 3). Sequence count (bp) of 74,158 (AGR-sunflower root from Itsoseng), 58,131 (AGS-sunflower stem from Itsoseng), 48,763 (BGR-sunflower root from Lichtenburg) and 56,387 (BGS-sunflower stem from Lichtenburg) and the sequence read count of AGR – 2,497, AGS – 1,507, BGR – 1,566 and BGS – 1,744 after quality control were recorded across the sampling sites. The number of reads generated for each sample that describes the bacterial community structure is presented by the rarefaction curve. Based on the saturation level, the maximum bacterial richness was found to be higher in AGR (sunflower root from Itsoseng) (Figure 2). The rarefaction depth for each sample across the sites, i.e. reads per species include, AGR (70,001 per 1265.5136), AGS (56,305 per 637.2572), BGR (46,781 per 784.1046), and BGS (56,305 per 637.2572), respectively.

**Table 3.** Statistical results for 16S rDNA sequencing

Sample information	TNS	SRAQC	CCEL
**AGR**	74,158	2,497	0
**AGS**	58,131	1,507	2
**BGR**	48,763	1,566	0
**BGS**	56,387	1,744	2

**Key**: AGR – root samples from Itsoseng, AGS – stem samples from Itsoseng, BGR = root samples from Lichtenburg, BGS – stem samples from Lichtenburg, TNS – total number of sequences, SRAQC – sequence read after quality control, CCEL – count of N characters exceeds the limit.

### 3.3 Endophytic bacterial composition and community structure at the phylum level

**Figure 3.**
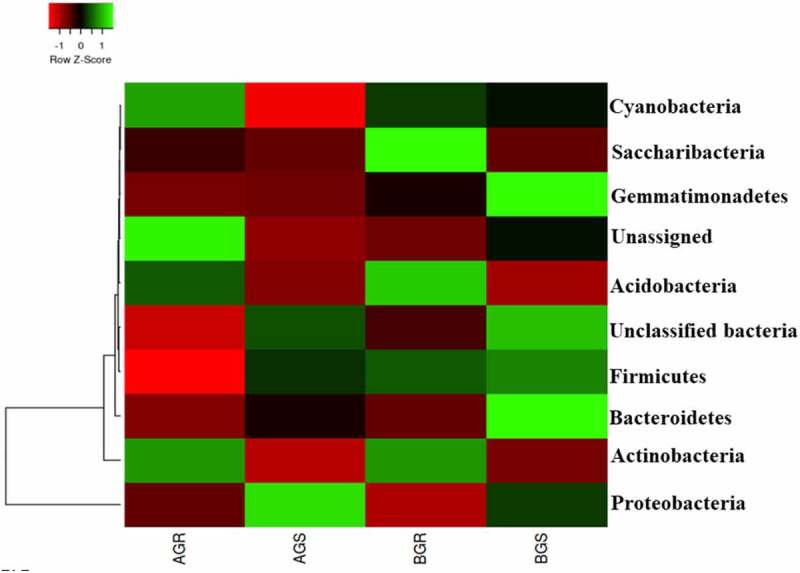
Taxonomic classification of bacterial phyla from roots and stem of growing sunflower Key: AGR = root samples from Itsoseng, BGR – root samples from Lichtenburg, AGS – stem samples from Itsoseng, BGS – stem samples from Lichtenburg.

**Figure 4.**
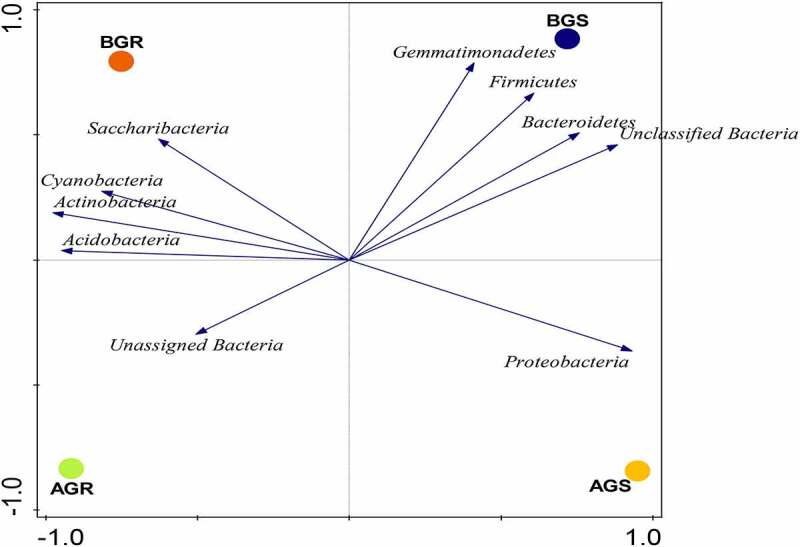
PCA plot of endophytic bacterial phyla associated with growing sunflower. The observed variance is represented on Axis 1 (88.5%) and Axis 2 (10.1%). Key: AGR = root samples from Itsoseng, BGR – root samples from AGS – stem samples from Itsoseng, Lichtenburg, BGS – stem samples from Lichtenburg.

In this study, the dominant endophytic bacteria phyla with higher richness in the samples across the sites were selected. The sunflower roots across the sites were dominated by Saccharibacteria and Acidobacteria in BGR (root samples from Lichtenburg), and unassigned bacteria in AGR (root samples from Itsoseng). Interestingly, Proteobacteria, Gemmatimonadetes, Bacteroidetes, and unclassified bacteria were dominant in AGS (stem samples from Itsoseng) and BGS (stem samples from Lichtenburg), respectively (Figure 3). However, their distribution across the plant organs at the phylum level was significantly different (*p* < .05) from each other as shown in (Table 2). The distribution of identifiable bacterial phyla across the two sites was more diversely distributed in root and stem samples from Lichtenburg (BGR and BGS) than the samples from Itsoseng. The distribution of bacterial genera in the root and stem across the collection sites was revealed in the PCA plot with Axis 1 explained 88.5% and Axis 2 explained 10.1% variance, respectively (Figure 4).

The vector distance of PCA revealed the most abundant bacterial phyla in each habitat (i.e., phyla on the longest distance length of PCA). Using the vector distance as a pointer, it is evident that Saccharibacteria, Cyanobacteria, Actinobacteria, and Acidobacteria dominated BGR, Gemmatimonadetes, Firmicutes, Bcateroidetes and unclassified bacteria dominated BGS, while unassigned bacteria and Proteobacteria were found dominant in AGR and AGS, respectively. The selection of bacterial phyla for PCA and PCoA plots was based on the level of significance (Table 2).

### 3.4 Endophytic bacterial diversity indices across the sampling locations

**Figure 5.**
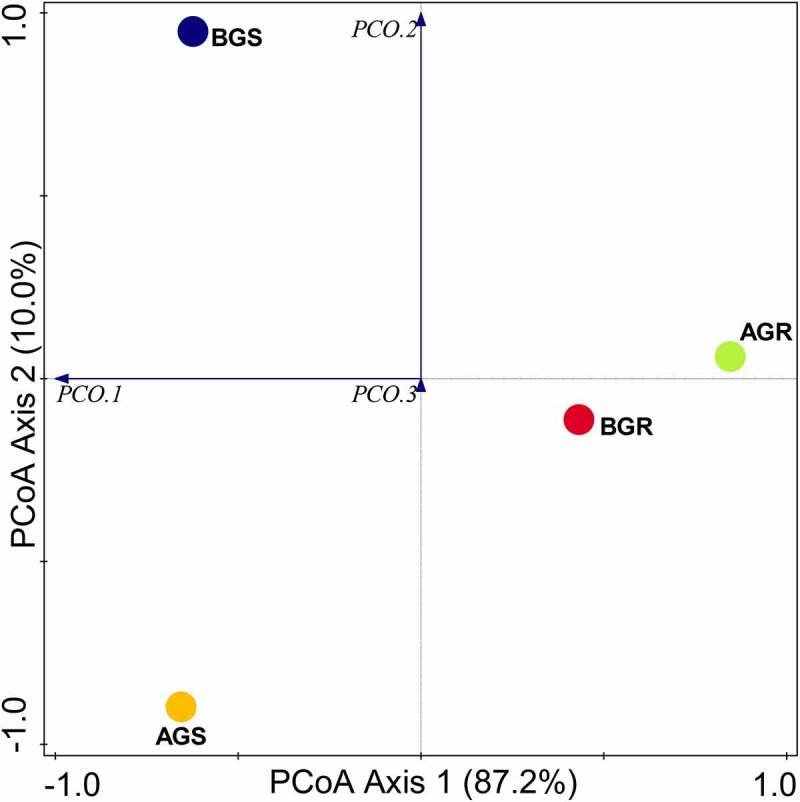
PCoA plot of bacterial phyla. Key: AGR = root samples from Itsoseng, AGS – stem samples from Itsoseng, BGR – root samples from Lichtenburg, BGS – stem samples from Lichtenburg.

Bacterial diversity evaluations using Evenness, Shannon, and Simpson indexes at the phylum and class levels were significantly different (*p* < .05), while no significant difference (*p* > .05) was observed in bacterial diversity at the genus level (Table 2). The PCoA analysis of endophytic bacteria using Bray–Curtis distance is shown in (Figure 5). Subsequently, the PCoA graph revealed significant differences in the endophytic bacterial composition in the BGS (stem sample from Lichtenburg) compared to other samples, namely, AGR (root sample from Itsoseng), AGS (stem sample from Itsoseng), and BGR (root sample from Lichtenburg) across the sites. ANOSIM (analysis of similarities) report showed a significant difference in the root and stem endophytic bacterial communities across the sampling sites with *p* = .01 and R = 0.585.

### 3.5 Endophytic bacterial distribution and community structure at the class level

**Figure 6.**
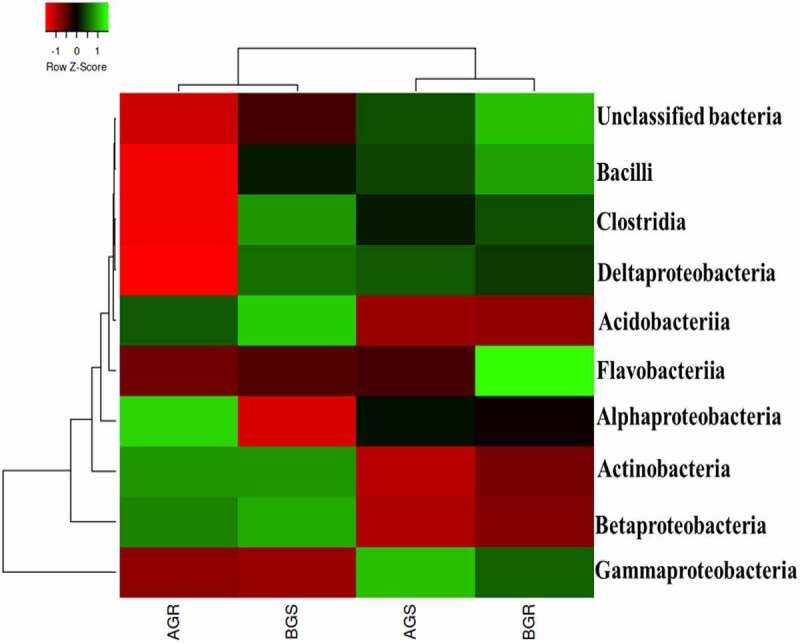
Taxonomic classification of bacterial distribution at a class level from the roots and stem of growing sunflower. Key: AGR = root samples from Itsoseng, AGS – stem samples from Itsoseng, BGR – root samples from Lichtenburg, BGS – stem samples from Lichtenburg.

The representative amplicon sequences from sunflower endophytic bacteria used in this study were compared using the SILVA database for the taxonomic assignment. The taxonomic classification of bacterial at the class level from the root and stem of growing sunflower, as shown in (Figure 6) revealed noticeable variations in the bacterial richness across the sites. Flavobacteriia was most dominant in BGR (root sample from Lichtenburg).

The predominance of Alphaproteobacteria and Actinobacteria in AGR (root sample from Itsoseng) were observed. AGS (stem sample from Itsoseng) and BGS (stem sample from Lichtenburg) were dominated with Gammaproteobacteria, and Betaproteobacteria, Acidobacteria, Clostridia, and Actinobacteria. Furthermore, Bacilli and unclassified bacteria were found dominant in BGR (root sample from Lichtenburg). A significant difference (*p* < .05) was observed in the distribution of endophytic bacterial isolates across plant organs at the class level (Table 2). Across the sites, Gammaproteobacteria exhibited a high relative abundance of 39.2% in AGR (root sample from Itsoseng), 73.59% in AGS (stem sample from Itsoseng), 63.94% in BGR (root sample from Lichtenburg), and 38.10% in BGS (stem sample from Lichtenburg), respectively.

### 3.6 Endophytic bacterial distribution and community structure at the genus level

**Figure 7.**
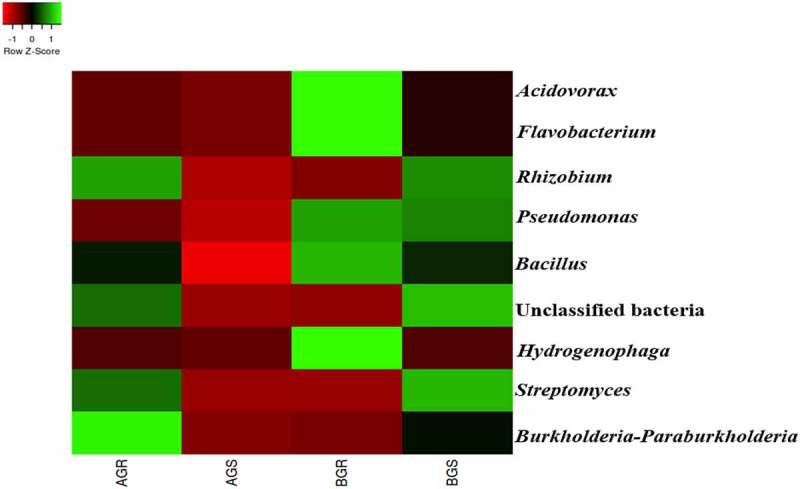
Taxonomic classification of bacterial genera from roots and stem of growing sunflower. Key: AGR = root samples from Itsoseng, AGS – stem samples from Itsoseng, BGR – root samples from Lichtenburg, BGS – stem samples from Lichtenburg.

At the genus level, *Acidovorax, Flavobacterium, Pseudomonas, Bacillus*, and *Hydrogenophaga* were the most dominant in BGR (root samples from Lichtenburg) compared to other samples. Additionally, *Streptomyces* and unclassified bacteria were predominant in BGS (stem samples from Lichtenburg), while *Rhizobium* and *Burkholderia-Paraburkholderia* were detected only in AGR (root samples from Itsoseng) (Figure 7). *Bacillus* predominantly shows high percentage composition of 75.04% in BGR, followed by BGS with a percentage composition of 49.64%, then, 47.61% in AGR, while the least percentage composition of 0.84 was observed in AGS.

### 3.7 Environmental factors influence the endophytic bacterial community structure

**Figure 8.**
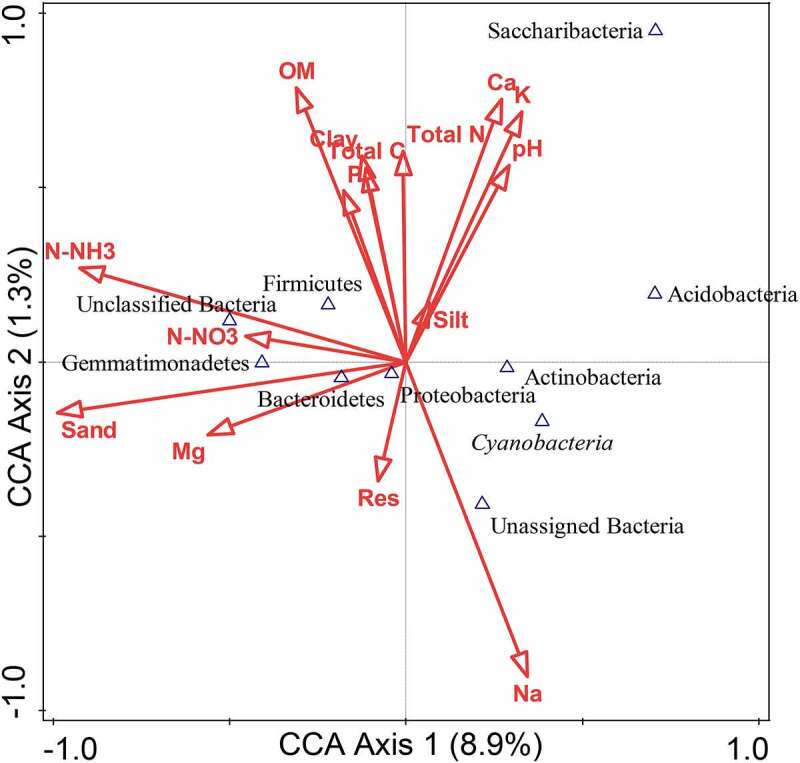
CCA plot of bacterial phyla and soil environmental variables.

The correlation between environmental variables on the distribution of bacterial endophytes at the phylum level was analyzed using the CCA. The soil parameters analysis (Table 2), was considered for the CCA plot (Figure 8). The results revealed that the bacterial community structure was induced by the soil parameters, with a CCA permutation test = 0.02.

Sand, calcium, potassium, sodium, organic matter, and N-NH_3_ showed prominent correlations with bacterial diversity than other environmental variables tested. Below the mid-point, Actinobacteria, Proteobacteria, Cyanobacteria, and unassigned bacteria positively correlated with Na, while negative correlation of Bacteriodetes with sand, Mg, and resistivity conductivity were observed compared to the soil parameters above the mid-point (Figure 8). The constraint environmental variables and testing with 499 random permutations were employed to deduce environmental variables and influence on bacterial diversity.

### 3.8 Predictive functional abundance information

**Figure 9.**
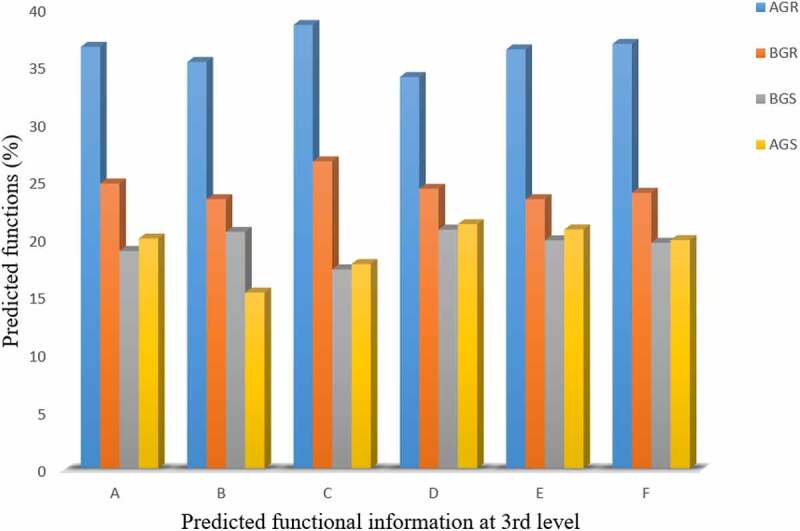
Percentage predicted functional information of bacteria in sunflower at the growing stage across different sites. Key: A – ABC transport, B – tryptophan metabolism, C – streptomyces metabolism, D – nitrogen metabolism, E – phosphonate and phosphinate metabolism, F – biosynthesis of siderophore group non-ribosomal peptides, AGR = root samples from Itsoseng, AGS – stem samples from Itsoseng, BGR – root samples from Lichtenburg, BGS – stem samples from Lichtenburg.

The predictive functions of endophytic bacterial across the sites were analyzed using PICRUSt. The predicted functional information of bacterial communities at a different level from each cluster is presented in (Table 4). At level 1 selection, bacterial possesses predictive functional information, such as cellular metabolism, and environmental information processing relating to plant growth promotion. The predicted functions revealed at third-level classification include ABC transport, tryptophan metabolism (a precursor for indole-3-acetic acid production), streptomycin biosynthesis (antibiosis), nitrogen metabolism (nitrogen fixation potential), phosphonate and phosphinate metabolism, and biosynthesis of siderophore and other secondary metabolites (Table 4 and Figure 9). High-predicted functional profiling of bacteria in the root samples collected from Itsoseng was recorded across the sites, while a lower tryptophan metabolism rate of 15.23% was recorded from the bacteria in stem samples from Itsoseng (Figure 9).

**Table 4.** Predicted functions of bacteria in sunflower at the growing stage

Predicted metabolism (1st level)	Activity (2nd level)	Predicted function (3rd level)
**Environmental information processing**	Membrane transport	ABC transport
**Metabolism**	Amino acid	Tryptophan metabolism
	Biosynthesis of other secondary metabolites	Streptomycin biosynthesis
	Energy metabolism	Nitrogen metabolism
	Metabolism of other amino acids	Phosphonate and phosphinate metabolism
	Metabolism of terpenoids and polyketides	Biosynthesis of siderophore group non-ribosomal peptides

## 4. Discussion

In a bid to understand plant endosphere biology, we present a next-generation sequencing approach to determine endophytic bacteria community structure in the root and stem of sunflower at the growing stage. In our view, this is the first report on the use of Illumina-based sequencing for determining bacterial community structure in sunflower cultivated in Southern Africa. Based on the farm history across the sites, we hypothesized that the diversity of bacteria in the sunflower endosphere may be influenced by soil parameters and agricultural practices (mono-cropping, and mixed cropping, as well as the use of chemical fertilizer and organic manure), which justify the choice for the sampling sites. The use of chemical fertilizer and organic manure in boosting soil nutrients and plant growth, on the other hand, may induce a shift in the soil parameters and bacterial community structure in its entirety.^43^ Research findings on the use of next-generation amplicon-based approach have been employed in determining bacterial diversity in rice, soybean, sunflower, and melon with success.^44–47^

In this study, dominant bacterial phyla, such as Saccharibacteria, Gemmatimonadetes, Acidobacteria, Bacteriodetes, and Proteobacteria were identified from the root and stem of sunflower at the growing stage. The presence of these bacteria might be due to their affinity to form a community within the sunflower endosphere. Similar bacterial phyla have been reported in the root, stem, and leaf endosphere of maize and peony.^19,48^ The unclassified bacteria phyla identified in the AGR (root sample from Itsoseng) and BGS (stem sample from Lichtenburg) may create insights for further research in determining their novel identity and exploration for agricultural and industrial use. The bacteria phyla identified in this study corroborate the findings of^49^, who reported identifiable endophytic bacteria phyla, Acidobacteria, Firmicutes, Bacteroidetes, Actinobacteria and Proteobacteria from the root of three species of medicinal licorices (*Glycyrrhiza uralensis, Glycyrrhiza glabra*, and *Glycyrrhiza inflata)* grown on plowed farmlands in China. The population of Acidobacteria, Bacteriodetes, and Proteobacteria in sunflower samples have been reported to contribute to plant growth promotion and health sustainably.^11^ The higher bacterial diversity in BGR and BGS can be linked to soil nutritional profile, organ differentiation, geographical location, and farming practices.^11^ Similarly, the lower bacteria diversity in AGR (root sample from Itsoseng) and AGS (stem sample from Itsoseng) can be attributed to root differentiation, low bacterial metabolic activity, weak infiltration from the external root environment, prevailing environmental factors, and agricultural practices.^50^ A difference in the relative abundance of endophytic bacteria community composition between roots and stems of sorghum plants has been reported by ^51^. Additionally, the effect of different agricultural practices and climatic conditions have been reported to influence the distribution of endophytic bacteria communities in the root endosphere.^52^ Interestingly, the difference observed in the bacterial community in the root and stem of sunflower at the growing stage in Lichtenburg compared to the stem of sunflower at the growing stage in Itsoseng validate the hypothesis of this study on the effect of crop rotational and mixed farming system on the bacterial diversity under different agricultural practices.

In this study, the diversity indices at phylum and class level showed a significant difference in the bacterial distribution across sampling sites and this can further explain how crop rotation systems and mixed cropping systems showed higher bacterial diversity in Lichtenburg than the mono-cropping system in Itsoseng. The crop rotation system in maintaining stable biodiversity and activities of endophytic bacteria has been documented by^53^. The successional mixed cropping system can enhance nutrient bioavailability and endo-rhizosphere competence, and this may selectively reintroduce different plant growth-promoting bacteria into the soil.^54^

The bacteria class identified in this study, such as Flavobacteriia in BGR (root sample from Lichtenburg) has not been documented in the root of any oilseed crops, thus revealing its bioprospecting in agriculture. Nevertheless, a study by^55^, revealing dominant Gammaproteobacteria and Flavobacteriia in the root of *Salicornia europaea* has been documented. The identifiable Gammaproteobacteria from the stem of sunflower [AGS] corroborate with^56^, who reported similar bacterial phyla from the leaf of tomato plants. Interestingly, the diverse classes of endophytic bacteria in the sunflower root can underline their significance in agriculture in enhancing plant growth.

At the genus level, the dominant *Acidovorax, Flavobacterium, Pseudomonas, Bacillus*, and *Hydrogenophaga* in BGR (root sample from Lichtenburg) compared to other samples may suggest agricultural importance of this bacterial genera. The agricultural importance of *Pseudomonas, Rhizobium*, and *Streptomyces* identified in this study may suggest their future exploration as bioinoculants in enhancing plant growth and control of plant pathogens. The biological relevance of *Pseudomonas* and *Bacillus* isolated from a medicinal plant *Echinacea purpurea* and their contribution has been reported in sustainable crop production and plant health.^57^ Due to the scanty information on the identification of plant growth-promoting endophytic bacteria genera from sunflower using amplicon sequencing, results from this study can serve as a model in future studies of endophytic bacteria associated with sunflower and other oilseed crops.

Subjecting agricultural soils to long-term fertilization may cause a change in the nutritional profile and bacterial diversity.^43^ In this study, the analyzed soil parameters showed a significant correlation to bacteria diversity. Sodium, organic matter, and ammonium nitrate best explain the bacterial diversity across the sites. The influence of these factors on bacteria diversity, however, may shape the bacterial structure and soil selection for agricultural purposes. The influence of sodium, phosphorus, magnesium, and potassium on the distribution of beneficial endophytic bacteria in the root of wheat has been documented.^58^

pH is one of the key determinant factors influencing bacterial community structure in the soil.^59^ The pH values of sunflower rhizosphere soil ranged from 6.37 to 7.06 and this corroborates with the findings of^60^, who reported pH values, 6.0, 6.5, 6.6, and 5.8 on sunflower rhizosphere soil collected from four different locations in South Africa. The influence of soil pH and organic matter has been reported to influence bacteria community structure in plants.^61,62^ From this study, the soil parameters assessed and their positive influence on bacterial distribution across the sites can influence bacteria potential in the soil. Hence, emphasis on the use of 16S rRNA amplicon metataxonomics in assessing diverse endophytic bacterial communities in sunflower can be promising in agriculture sustainably. In addition, endophytic bacteria identified in this study may play significant roles in recycling soil nutrients, improving plant growth, and soil health. Furthermore, the sunflower endophytic bacteria community structure as confirmed in this study and their higher distribution in root compared to stem reveal similar predictive functional attributes in the below-and-above ground levels.

The bacterial predictive functions at third-level classification include ABC transport, tryptophan metabolism (a precursor for indole-3-acetic acid production), streptomycin biosynthesis (antibiosis), nitrogen metabolism (nitrogen fixation potential), phosphonate and phosphinate metabolism, and biosynthesis of siderophore and other secondary metabolites, and these predicted functions can be linked to the interdependence of endophytic bacteria with the host plants across the sites (Itsoseng and Lichtenburg). The predicted functional traits of endophytic bacteria across the sites may underline their effects on plant growth promotion and enhancing soil fertility. Production of certain secondary metabolites has been attributed to the Actinobacteria and Acidobacteria found in the root-soil environment, which enhances their ability to metabolize organic substrates and controlling plant pathogens.^63–65^ Other predicted functions involved indole-3-acetic acid and siderophore production, antibiotic synthesis, and phosphate solubilization may enhance plant growth and health through nitrogen fixation, root development, the bioavailability of soil nutrients for plant uptake, and synthesis of biocontrol agent against phytopathogens.^66^ In line with this study, the predicted functional analysis of endophytic bacteria associated with sunflower root and stem corroborate the findings of^67^, who reported similar potential functions exhibited by endophytic bacteria associated with flowering and nonflowering moso bamboo (*Phyllostachys edulis*). The predicted functional reports on endophytic bacteria colonizing the sunflower endosphere have not been documented. Hence, the observed predicted functions exhibited by endophytic bacteria in this study can serve as a guide for future studies.

## 5. Conclusion

In the present study, 16S rRNA gene amplicon sequencing was employed to evaluate the bacterial communities in the root and stem of sunflower at the growing stage across the sampling sites. The results obtained at the phyla level revealed a significant difference in bacterial diversity across the sites, with the most dominant in sunflower root collected from Lichtenburg. The predominance of unclassified bacteria in the stem sample from Lichtenburg suggests further studies in devising culturable means for their characterization and detection of novel traits that can be harnessed as bioinoculants in developing eco-friendly agriculture. Environmental variables showed positive and negative influences on bacteria diversity across the sites. The predicted functions of these bacteria suggest their agricultural importance, which can be explored in developing bioproducts as an alternative to chemical fertilizer. Promisingly, due to the economic value of this sunflower, it is recommended to use culture-dependent techniques and practicable *in vitro* seed inoculation and planting under greenhouse to further experiment with the potential of endophytic bacteria on sunflower plants.

Furthermore, understanding plant-associated microbes under different farming systems will help determine their functional roles in plant nutrition, growth, and health, although information on sunflower-associated endophytic bacteria is limited. Interestingly, this study provides clear evidence on the influence of crop rotation, agricultural practices, and soil parameters on the bacteria diversity in sunflowers from the two sites. In conclusion, this study may serve as a model in future endosphere biology under different agricultural systems to enhance plant performance, comparing the plant growth stages, locations, and plant organs.

## Data Availability

The sequence was deposited in the Sequence Read Archive (SRA) of the National Center for Biotechnology Information (NCBI) with Bioproject accession numbers https://www.ncbi.nlm.nih.gov/bioproject/PRJNA673781 and https://www.ncbi.nlm.nih.gov/bioproject/PRJNA673791.
